# Immunomodulatory LNP-mRNA therapeutics: The next phase of precision immuno-engineering

**DOI:** 10.1016/j.omtn.2026.103044

**Published:** 2026-08-12

**Authors:** Krupal Patel, Colt Egelston, Tristan Scott

**Affiliations:** 1 Department of Surgery, City of Hope National Medical Center, 1500 E. Duarte Road, Duarte, CA 91010, USA; 2 Division of Head & Neck Surgery, Department of Surgery, City of Hope National Medical Center, 1500 E. Duarte Road, Duarte, CA 91010, USA; 3 Department of Immuno-Oncology, Beckman Research Institute, City of Hope, 1500 E. Duarte Road, Duarte, CA 91010, USA; 4 Center for Gene Therapy, City of Hope, Beckman Research Institute and Hematological Malignancy and Stem Cell Transplantation Institute at the City of Hope, 1500 E. Duarte Road, Duarte, CA 91010, USA

**Keywords:** lLipid nNanoparticles, immunomodulators, TMEIntroduction

## Introduction

Immunosuppressive tumor microenvironments (TMEs) are often associated with aggressive disease, increased metastasis, and reduced response to therapies like immune checkpoint inhibitors (ICIs).^1^ Stereotypically, suppressive TMEs have low infiltration of T cells (cytotoxic CD8^+^ or Th1-dominant CD4^+^) and natural killer (NK) cells, with a high presence of anti-inflammatory myeloid cells (M2-like macrophages) and T regulatory cells (Tregs).^1^ In T cell-inflamed TMEs, chronic activation by tumor antigens, including neoantigens, can promote exhaustion among tumor-reactive T cells.^2^ The immunosuppressive TME can be reversed by promoting (1) recruitment of proinflammatory and cytotoxic immune cells, or (2) reversal of exhausted, immunosuppressive phenotypes of infiltrating lymphocytes and myeloid cells, as well as restoring immune surveillance, tumor control, remission, and immunological memory.

Cytokines are small, secreted proteins essential for the activation and growth of immune cells and crucial in controlling cell signaling pathways. However, the systemic administration of recombinant cytokine proteins has narrow therapeutic windows with high dose-limiting toxicity. Systemic IL-2 toxicities include capillary leak syndrome, hypotension, and hepatic, renal, cardiac, and pulmonary dysfunctions requiring intensive care-level support.^3^ IL-15 applied systemically has a short half-life of approximately 2.5 h. In a phase 1 clinical trial, daily infusions up to 12 days had similar side effects as IL-2, although with lower severity and no observed cardiac arrythmias or ischemia.^3^ The daily infusions are a significant barrier for patients. Thus, new therapeutic approaches are needed to overcome the narrow therapeutic window and dose-limiting toxicity of immunomodulatory strategies.

## Redefining immunomodulation with lipid nanoparticles

Lipid nanoparticles (LNPs) that encapsulate RNA encoding cytokines present several advantages over recombinant proteins: (1) formulations can be modified to target certain tissues and cells (“tropism”), (2) different effector cytokines can be combined in one injection to affect multiple immune cells and pathways simultaneously, and (3) LNPs have intrinsic enhanced permeability and retention (EPR) within tumor tissues.^4^ Generally, LNPs consist of four lipids: a “helper” lipid, ionizable lipid, cholesterol, and a polyethene glycol (PEG) derivative that encapsulate a coding RNA. Two main formats of RNA have been used: mRNA and self-replicating RNA (srRNA), with srRNA allowing limited rounds of “self-replication” to increase cytokine expression. The non-immunogenic LNP allows repeat dosing, while the RNA format enables a predictable “burst” of cytokine expression that minimizes systemic toxicity associated with immune hyperactivation. To date, these formats have been used to express several cytokines, with a range of roles in TME restructuring (see Table 1).

**Table 1:** Immunomodulators expressed for an LNP-RNA format in preclinical studies

Factor	Function in immune invigoration or TME remodeling
IL-12	stimulate macrophages, monocytes, and DCs to induce the proliferation of cytotoxic T and NK cells and interferon γ (IFN-γ) production
IL-27	anti-inflammatory that improves T cell survival and induces memory CD8^+^ T cell
GM-CSF	induces the differentiation of granulocytes, monocytes, and macrophages
4-1BB	induces CD8^+^ T cells following TCR stimulation, enhancing proliferation and cytotoxic production
IL-7	induces naive CD8^+^ T cell differentiation into a memory phenotype; protection of T cells against adenosine-mediated immunosuppression
IL-21	promotes T and NK cell secretion of IFN-γ
OX40L	regulates cytokine production from T cells, antigen-presenting cells, and NK cells
IL-36γ	acts on professional APCs and T cells to inhibit tumor growth
IL-23	positively modulates the bridge between innate and adaptive responses
IL-15	central role in innate and adaptive immunity

## The clinical landscape of LNPs and immunomodulators

Several LNP formulations have entered the clinic, with a focus on the well-established IL-12 that stimulates myeloid cells to induce the proliferation of cytotoxic lymphocytes (CTLs; Tables 1 and 2). In recent trials, factors other than cytokines have been used such as the immune co-factor OX40L that has been used alone or in combination with the cytokines IL-24 and IL-36γ (Table 2). Moreover, correlative studies have shown that the anti-tumor effects by ICIs are associated with higher T cell densities and markers such as PD-L1 and signatures of pro-inflammatory interferon γ.^5^ LNP-cytokine formulations can stimulate these effects and are often combined with ICIs to enhance their efficacy (Table 2).

**Table 2:** Clinical landscape of immunomodulators with LNPs and RNA

Payload	Cancer	Product name	Adjuvant	Sponsor	Trial ID	Status	Phase	Last update	Location
OX40L/IL-23/IL-36γ mRNA	advanced or metastatic solid tumor malignancies or lymphoma	mRNA-2752	durvalumab (±)	Moderna therapeutics	NCT03739931	completed	phase 1	8/15/2025	USA, Australia, Israel
OX40L/IL-23/IL-36γ mRNA	high-risk ductal carcinoma	mRNA-2752	pembrolizumab (±)	Laura Esserman (Moderna therapeutics)	NCT02872025	active, not recruiting	phase 1	1/30/2025	USA
IL-12 mRNA	advanced solid tumors	MEDI1191	durvalumab (±)	MedImmune LLC	NCT03946800	completed	phase 1	10/2/2024	USA
OX40L mRNA	advanced relapsed/refractory solid tumor malignancies or lymphoma	mRNA-2416	durvalumab (±)	Moderna therapeutics	NCT03323398	terminated	phase 1/2	7/31/2024	USA
IL-12 self-replicating RNA	recurrent or progressive high-grade glioma	JCXH-211	monotherapy	Duke University	NCT06468605	withdrawn	phase 1	7/23/2025	no data
IL-12 self-replicating RNA	malignant solid tumors	JCXH-211	monotherapy	Immorna Biotherapeutics, Inc.	NCT05539157	unknown status (active, not recruiting)	phase 1	4/4/2023	USA
IL-12 self-replicating RNA	malignant solid tumors	JCXH-211	monotherapy	Immorna Biotherapeutics, Inc.	NCT05727839	unknown status (active, not recruiting)	phase 1	4/4/2023	China
IL-12 self-replicating RNA	malignant solid tumors	JCXH-211	toripalimab (+)	Immorna Biotherapeutics, Inc.	NCT06781125	not yet recruiting	phase 1	1/17/2025	no data
IL-12 mRNA	advanced solid tumors	ABOD2011	monotherapy	Cancer Institute and Hospital, Chinese Academy of Medical Sciences	NCT05392699	recruiting	phase 1	6/22/2022	China
IL-12 mRNA	advanced solid tumors	ABO2011	toripalimab (±)	Suzhou Abogen Biosciences Co., Ltd.	NCT06088004	recruiting	phase 1/2	8/22/2025	China
IL-12 self-replicating RNA	advanced solid tumors	STX-001	pembrolizumab (±)	Strand Therapeutics Inc.	NCT06249048	recruiting	phase 1/2	10/29/2025	USA, Australia

Moderna’s mRNA-2752, an LNP combination of OX40L, IL-23, and IL-36γ with distinct mechanisms of action (see Table 1), was injected intratumorally in advanced solid cancers.^6^ mRNA-2752 met phase 1 safety endpoints and showed responses with tumor reduction in patients with melanoma and triple-negative breast cancer.^6^ Furthermore, biomarkers of pro-inflammatory cytokines were detected after treatment in the plasma and tumor after 6–24 h with higher T cell immune infiltration.^6^ Higher immune responses were associated with clinical benefit.^6^ Intratumoral injection of mRNA-2752 and the pembrolizumab in high-risk ductal carcinoma showed promising effects in a small female patient population.^7^ Additionally, MEDI1191,^8^ an IL-12 locally administrated with LNPs, was tested mostly in melanoma, but also in pancreatic, colorectal, gastric, anal, and neuroendocrine cancers, and, likewise, had a favorable safety profile, as dose-limiting toxicity was not reached. Although clinical data are currently limited, intriguingly, there were observed reductions in tumor size in untreated tumors in patients for mRNA-2752,^6^ mRNA-2416,^9^ and MEDI1191,^8^ suggesting a broader immune effect beyond the local treated site, but further studies are needed.

Recent clinical setbacks warrant reflection on trial outcomes with a single cytokine, mRNA-2416 (LNP-OX40L mRNA), in combination with durvalumab. Although safety criteria were met and some efficacy was noted, the study was terminated due to not meeting the overall efficacy endpoints (NCT03323398). A notable case in the trial was serous fallopian/ovarian cancer with stable disease regressing almost completely, with surrounding non-injected lesions also showing anti-tumor effects.^9^ Although promising efficacy endpoints were met in advanced cancers within pre-treated patients, a large fraction of advanced solid cancer was unresponsive to mRNA-2752^6^ and MEDI1191,^8^ even in combination with ICIs. Closer examination of the clinical data will shed important insights into the next generation of LNP-mediated immunomodulatory approaches and build on these early successes.

## Future considerations

The current trial landscape provides a rationale to pursue delivery of new LNP-delivered immunomodulators to treat solid cancers. IL-15 superagonists could benefit from local treatment with LNPs to reduce toxicities and show promising preclinical results with favorable toxicity profiles and will likely enter the clinical landscape.^10^ Other cytokines and combinations are currently being assessed in preclinical studies (Table 1).

Administration routes should also be considered. Direct-to-tumor approach allows for increased effective concentration of the immunomodulatory cytokines loco-regionally within the tumor tissue or in the tumor draining lymph nodes (TDLNs). Direct injection, however, has limitations when the lesions cannot be accessed due to anatomical barriers, or there is a high metastatic disease burden. Systemic administration may provide a more conventional clinical administration route. Recent preclinical studies for the intravenous administration of LNPs delivering IL-12 encoded by a srRNA (STX-003) has a favorable anti-tumor efficacy and safety profile.^10^ The route of administration may be dependent on the type of tumor, tumor location, and extent of tumor burden.

Understanding LNP biodistribution within the TMEs is critical. LNP injected locally may have a “tropism” for specific cell types, and the uptake and expression in tumor cells, stroma, and immune populations influence therapeutic outcomes. Yet, only a few studies have characterized the TME cellular tropism of LNPs.^4^ Novel LNP designs should take advantage of these cellular tropism characteristics to favorably restructure the TME. Another important consideration is the preclinical model systems to test LNPs. The complexity of solid cancers with dynamic and reactive TMEs warrants more careful consideration. Preclinical studies have relied on syngeneic mouse models of homogeneous melanoma, colon, or breast cancer, which may fail to capture the complexity of human immune interactions with heterogeneous patient tumors.^4^ Recent advances in humanized murine models combined with more representative patient-derived xenograft (PDX) tumors may hold promise in this area but requires further validation on how accurately the models will recreate the patients’ TME and predict therapeutic responses.

## Conclusion

LNP-mRNA technology has the potential to reshape TMEs and immunotherapy. New trials have started exploring intratumoral administration of LNPs in combination with ICIs, or cytokines that require long therapeutic windows to have an effect (Table 2). With demonstrated safety in early-phase trials with various immunomodulator payloads and emerging efficacy data, as well as tentative effects on distal tumors, the field is poised for significant clinical impact. Clear demonstrations of anti-tumor activity in late-stage trials are essential to sustain investment in LNP-mRNA therapeutics, and, in combination with other immunotherapies and cell therapies, LNP-mRNA platforms could significantly improve outcomes for patients with advanced cancers.

## Acknowledgments

This work was supported by National Institute of Health, National Cancer Institute on a P30-CCSG supplement award (NCI, P30CA033572-43S2), the Circle 1500 Grant Award, a P30 CCSG supplement award for Early-Stage Investigators (NCI, P30CA033572), Bristol-Myers Squibb Foundation - Robert A. Winn Career Development Award, Warren Family Head and Neck Cancer Research Fund, Bryan and Helga Isaacs Head and Neck Cancers Clinical Care Initiative. The content is solely the responsibility of the authors and does not necessarily represent the official views of the National Institutes of Health, Circle 1500 Award Board, or any of the other entities and foundations mentioned.

## Declaration of interests

The authors disclosure there are no conflicts of interest.
